# Research hotspots and trends in the application of electroencephalography for assessment of disorders of consciousness: a bibliometric analysis

**DOI:** 10.3389/fneur.2024.1501947

**Published:** 2025-01-27

**Authors:** Jiawen Chen, Yanhua Shi, Zhao Dong, Feng Xu, Mengyu Zhou, Jing Zhu, Jie Gao, Su Liu

**Affiliations:** ^1^Department of Rehabilitation Medicine, Affiliated Hospital of Nantong University, Nantong, China; ^2^School of Nursing and Rehabilitation, Nantong University, Nantong, China; ^3^Nanjing Vocational Health College, Nanjing, China; ^4^The Second People's Hospital of Nantong, Nantong, China

**Keywords:** disorders of consciousness, electroencephalography, bibliometric analysis, research trends, unresponsive wakefulness syndrome, minimally conscious state

## Abstract

**Objective:**

Disorders of consciousness (DoC) result from severe traumatic brain injury and hypoxia or ischemia of brain tissues, leading to impaired perceptual abilities. Electroencephalography (EEG) is a non-invasive and widely applicable technology used for assessing DoC. We aimed to identify the research hotspots in this field through a systematic analysis.

**Methods:**

Relevant studies published from January 1, 2004 to December 31, 2023 were retrieved from the Web of Science Core Collection database. The data were analyzed and visualized using CiteSpace, VOSviewer, and SCImago Graphica.

**Results:**

In total, 1,639 relevant publications were retrieved. The country with the highest number of publications was the United States, the most productive institution was Harvard University, the journal with the highest output was *Clinical Neurophysiology*, and the journal with the highest total number of citations was *Neurology*. The author with the most publications was Steven Laureys and the most common keyword was “vegetative state.”

**Conclusion:**

The field is undergoing rapid development, characterized by a proliferation of advanced technologies and an increased emphasis on international collaboration. The document offers an impartial perspective on the advancements of the research study for the benefit of the researchers.

## 1 Introduction

Disorders of consciousness (DoC) are characterized by impaired perception of the surrounding environment that often result from a traumatic brain injury and manifest as changes to focus, perception, memory, and cognition. According to Sniff's central circuit theory, DoC are associated with weakening of the central thalamus and subsequent reduced activities of the cortico-thalamic and thalamo-striatal circuits (1). Based on the behavioral responses of patients, DoC are clinically classified as coma, unresponsive wakefulness syndrome (UWS) and minimally conscious state (MCS). Patients with DoC frequently encountered in clinical settings often present with critical conditions in the acute phase and long-term prognoses widely vary, thus posing significant burdens to families and society (2, 3). Assessment of early impairment of brain function, prognosis, and selection of an adequate treatment strategy are particularly challenging. DoC are often misdiagnosed, which significantly impacts prognosis and treatment (4–6).

The diagnostic and treatment tools commonly used for DoC can be categorized into clinical behavioral examinations and objective examinations. Behavioral assessment is considered the “gold standard” for diagnosis and prognosis of patients with DoC, but the integrity and clinical utility have not been fully demonstrated (7). Neuroimaging and electrophysiological tools, such as electroencephalography (EEG), magnetic resonance imaging (MRI), positron emission tomography (PET), and transcranial magnetic stimulation (TMS) combined with EEG, are highly accurate and reliable for diagnosis of DoC (8–10). In our bibliometric analysis, we found that the number of studies related to EEG was significantly higher than that for other techniques. This led us to focus primarily on EEG to ensure a comprehensive and in-depth review.

EEG is a non-invasive, cost-effective, and readily accessible neuromonitoring tool for assessment, diagnosis, and prognosis of patients with DoC (11). Since repurposed in 1942 by W. Grey Walter as a practical diagnostic aid, EEG is commonly used for diagnosis and prognosis of patients with DoC in the early stages of recovery (12, 13). In 2020, the European Academy of Neurology affirmed the diagnostic and prognostic value of EEG reactivity to external stimuli in the publication “Coma and Other Disorders of Consciousness Diagnostic Guidelines” (3). In recent years, the application of EEG for assessment of DoC has received considerable attention. A prior study found that high-density EEG can improve localization accuracy (14). Moreover, EEG-based brain-computer interface systems can provide communication and control for DoC patients (15). High-frequency oscillations have garnered attention as an emerging diagnostic biomarker for epilepsy, although further validation is needed for clinical application. Additionally, electrical source imaging is an accurate and clinically useful multimodal tool for pre-surgical assessment of drug-resistant focal epilepsy (16).

Bibliometrics involves the application of mathematical and statistical techniques to quantify and analyze the volume of literature, thereby elucidating the developmental characteristics of the discipline. In light of the increased public interest in the recent advancements in this field, there exists a noticeable absence of corresponding bibliometric analysis. Consequently, we have undertaken the collection and analysis of pertinent literature to delineate the trajectory of EEG assessment of DOC. Our objective is to furnish enhanced guidance for clinical applications (17).

## 2 Materials and methods

### 2.1 Data collection and retrieval strategy

**Figure 1:**
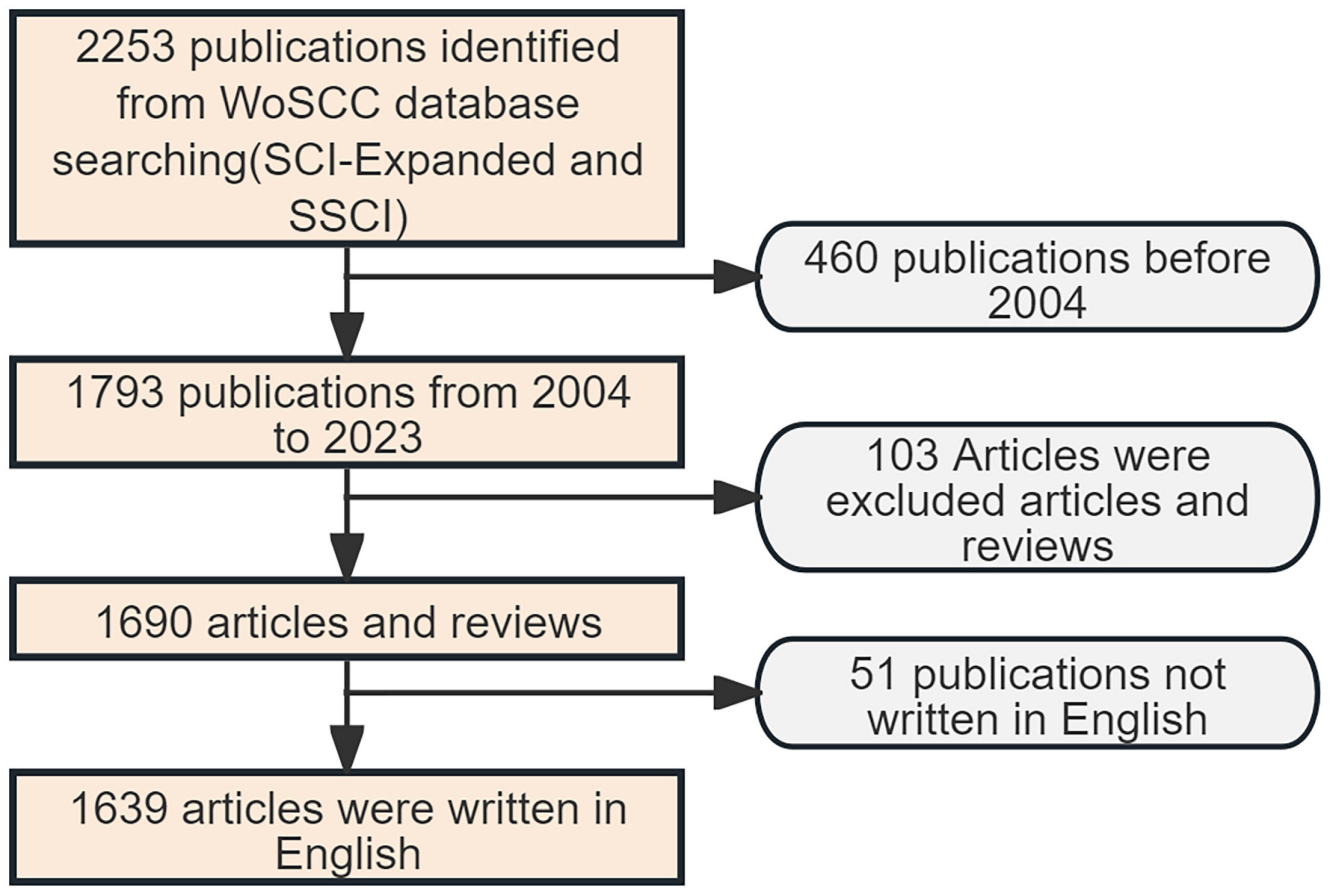
Flow chart of the literature review process.

Relevant studies published from January 1, 2004 to December 31, 2023 were retrieved from the Web of Science Core Collection database using the search terms (“unresponsive wakefulness syndrome” OR “minimally conscious state” OR “vegetative state” OR “disorders of consciousness” OR “coma”) AND (“EEG” OR “electroencephalography” OR “electroencephalogram”) (18). This time frame was chosen to emphasize recent advancements and applications of EEG in the assessment of DOC. To avoid bias, all English-language articles and reviews were downloaded on May 1, 2024. In total, 1,639 relevant publications were eventually acquired. The specific literature screening process is detailed in Figure 1.

### 2.2 Analytical tools and methodologies

Analysis was performed using CiteSpace V6.2 R6 (https://citespace.podia.com/) and VOSviewer (https://www.vosviewer.com/) with the SCImago Graphica (https://www.graphica.app/) as a supplementary tool. In our study, we utilized the metadata of the articles. This included essential information such as the title, authors, publication year, journal, and keywords, which allowed us to conduct our analysis effectively.

VOSviewer has excellent data processing capabilities, which allows for clear and intuitive presentation of the results. In this study, VOSviewer was utilized to identify countries, institutions, journals, authors, and articles, as well as co-occurrence and cluster analyses. The default settings of VOSviewer were applied (19).

CiteSpace is a powerful tool for visualizing and analyzing research trends within a discipline from a macroscopic perspective. In the present study, CiteSpace was used to create journal biplot overlays and clustering analyses of keywords, and conduct burst analyses of keywords and references. The specific parameters included a time slice of 1 year from January 1, 2004 to December 31, 2023. The node-type settings included keywords and cited literature. Based on the selection criteria, the top 50 articles for each time slice were identified. Pruning options (“pathfinder,” “pruning sliced networks,” and “pruning the merged network”) were also utilized. All other settings were set at default values (20–22).

SCImago Graphica is a robust visualization and analysis tool that can be utilized across various data display and analysis scenarios, enabling users to gain a more intuitive understanding of complex data (23).

## 3 Results

### 3.1 Analysis of publication volume, citation trends, and country metrics

**Figure 2:**
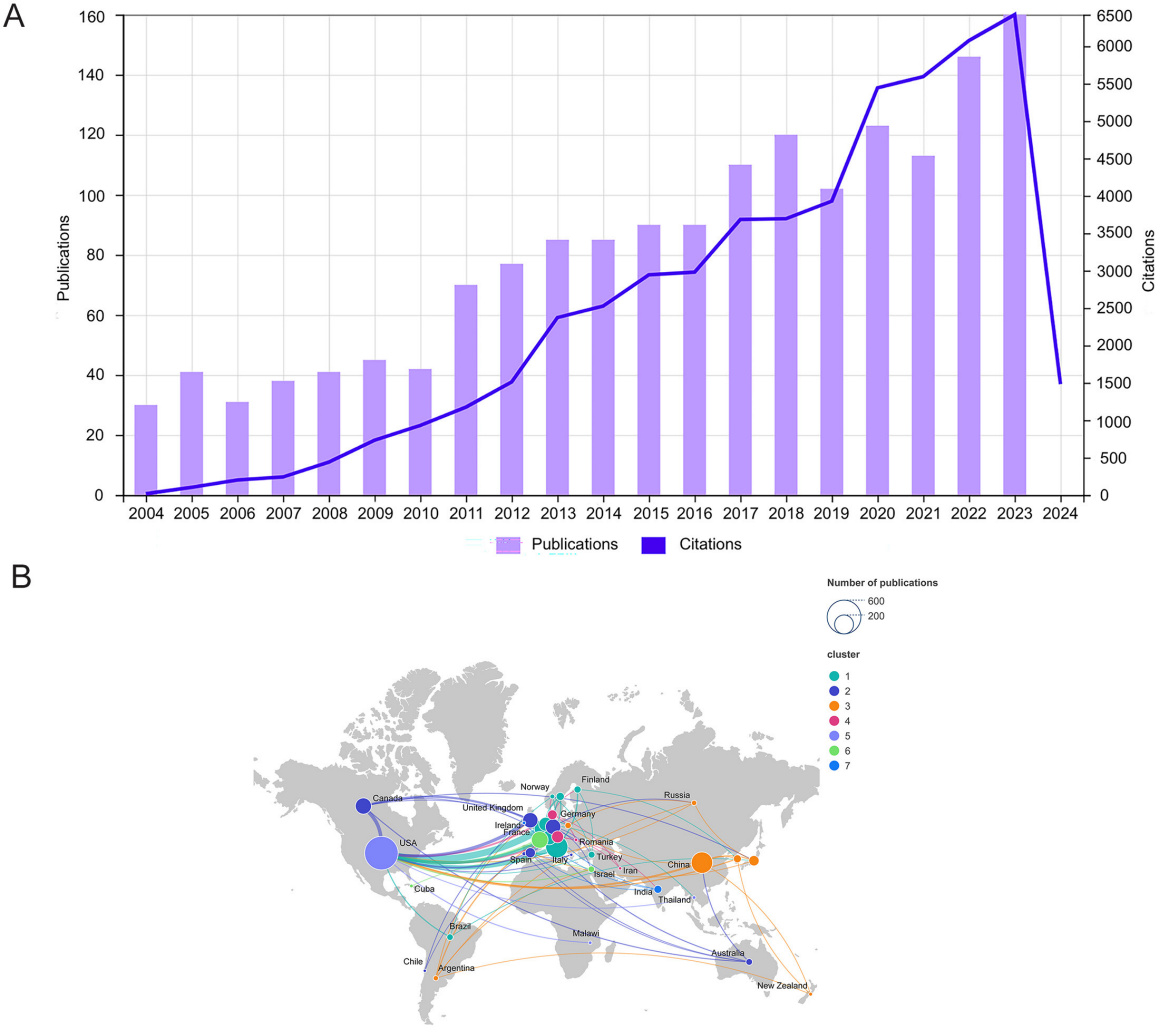
**(A)** Trends in annual publications and citations on the application of EEG for assessing DOC from 2004 to 2023. The bar chart illustrates the distribution of 1,639 publications from WoSCC over 20 years, while the line chart depicts the annual changes in the number of citations for these publications. **(B)** Cooperation between countries on a geographical map. Nodes represent countries, with their size corresponding to the number of publications. The upper right corner illustrates the node sizes for publication counts of 200 and 600. Lines connecting the nodes signify cooperation between countries, with the thickness of the lines indicating the frequency of collaboration.

The built-in functionality of the Web of Science Core Collection database was used to analyze the results of previous searches and create Figure 2A. The level of interest in the field was gauged by identifying trends in the number of publications. Over the past 20 years, the annual number of publications in the field remained relatively stable from 2004 to 2010, slightly increased from 2011 to 2018, and exhibited minor fluctuations between 2019 and 2023, reaching a 20-year peak in 2023 with 160 publications. The number of citations showed a similar trend. As depicted in the graphs of annual trends in the number of publications and citations, the potential of EEG for assessment of DoC has been increasingly scrutinized, and is expected to become a focus of future research.

The 1,639 relevant publications were from 75 countries. The top 10 countries based on the number of publications are listed in Table 1 and Figure 2B. The top three countries in terms of the number of publications were the United States (545, 33.3%), Italy (238, 14.5%), and China (221, 13.5%). The top three countries in terms of centrality ranking were the United States (0.47), Italy (0.23), and England (0.12). Higher centrality indicates more influence and significance. The SCImago Graphica and VOSviewer were utilized to assess cooperation among countries. Figure 2B illustrates the geographical distribution of each country, along with the volume of publications and the patterns of collaboration between countries. Nodes represent countries, with the size of each node reflecting the number of published articles. Different colors indicate distinct clusters, while the connections between nodes illustrate cooperation between countries, with the thickness of these connections representing the frequency of collaborative efforts. These countries had high thesis output and citation rates, and the research had significant impacts on neighboring countries. Cooperation is more pronounced among countries in closer geographical proximity, particularly in Europe, and is also increasing between continents. Through international collaboration, researchers can share data and methods to enhance the accuracy of EEG in diagnosing and prognosing DOC, foster the development of new technologies, and elevate the overall standard of global research.

**Table 1:** Top 10 most productive countries.

**Rank**	**Country**	**Count (%)**	**Citations**	**Average article citations**	**Centrality**	**H-index**
1	USA	545 (33.3%)	25,157	46.16	0.47	83
2	Italy	238 (14.6%)	9,340	39.24	0.23	53
3	China	221 (13.5%)	2,531	11.45	0.05	24
4	Belgium	147 (8.9%)	7,649	52.03	0.11	45
5	France	135 (8.2%)	5,219	38.66	0.08	38
6	Canada	126 (7.7%)	5,534	49.32	0.04	38
7	Germany	113 (6.9%)	3,904	34.55	0.12	34
8	England	106 (6.5%)	6,424	60.6	0.12	40
9	Switzerland	103 (6.3%)	6,265	60.83	0.05	38
10	Netherlands	92 (5.61%)	5,096	55.39	0.07	36

### 3.2 Institutional analysis

**Figure 3:**
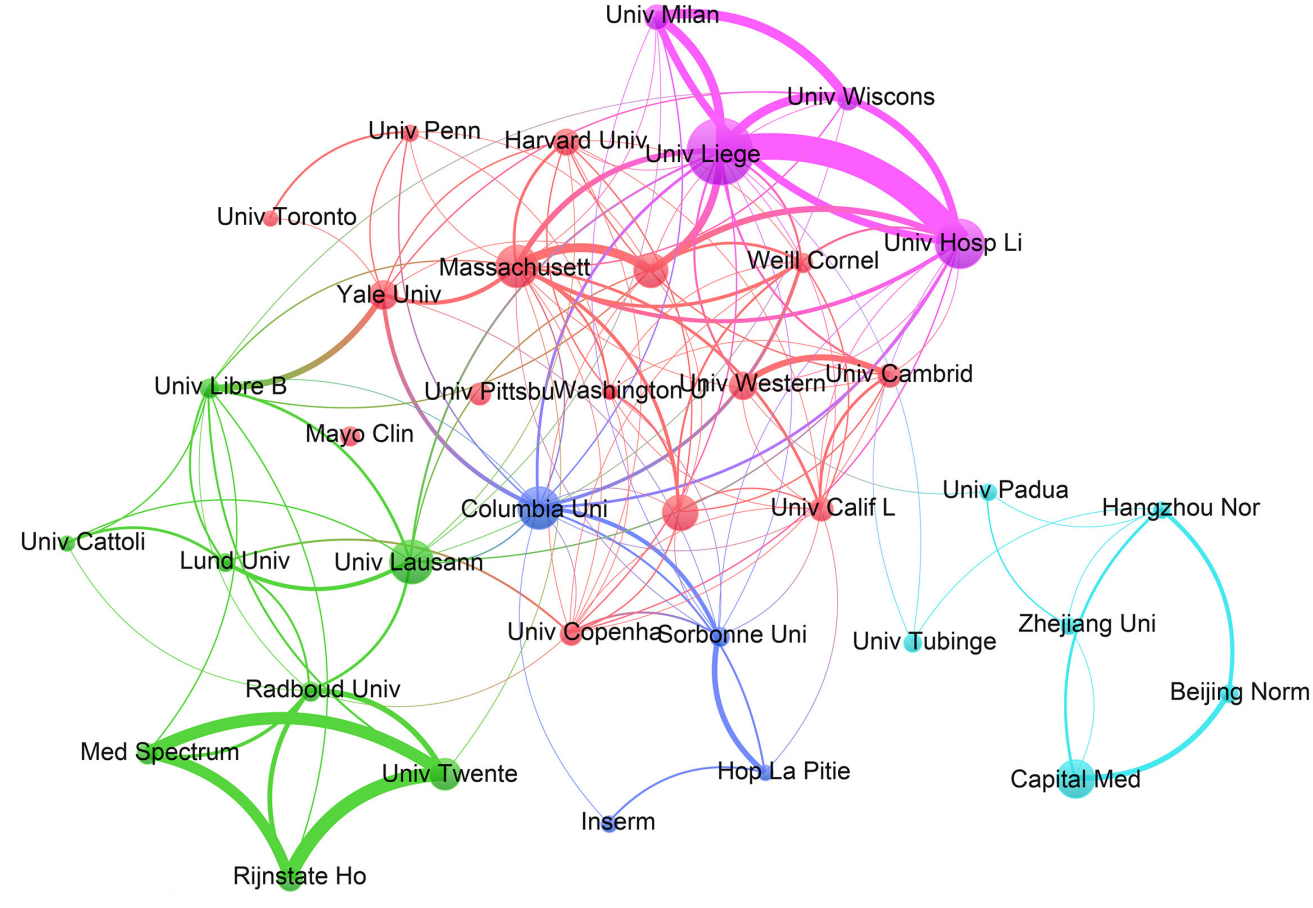
Inter-agency partnerships. The size of the circle shows the number of articles originating from the institution. The thickness of the connecting lines shows the strength of cooperation among institutions. Agencies that cooperate more frequently form color-coded clusters.

The standing of an institution in the field is reflected by annual output and production quality. The top 10 institutions ranked by the number of publications are listed in Table 2. Of these, four of the most productive institutions were located in the United States, three in France, two in Switzerland, and one in Belgium. The top three institutions in terms of the number of publications were Harvard University (112, 6.9%), Institut National de la Sante et de la Recherche Medicale (Inserm) (91, 5.6%), and the University of Liege (91, 5.6%). The top-ranked institution, Harvard University, received 6,013 citations and has an h-index of 37. The most influential institutions in terms of centrality, particularly the impact on the role of EEG for assessment of DoC, were Johns Hopkins University (0.12), Harvard University (0.11), and the University of Liege (0.09). Figure 3 clearly illustrates the dynamics of inter-agency cooperation. Larger nodes indicate a higher volume of outgoing documents, while thicker lines represent more frequent collaboration between agencies. Nodes of the same color denote a specific cluster. Institutional cooperation is apparently geographically influenced, we observed that most items within a cluster originate from a specific region. Institutions in Europe and the United States tend to prefer international collaboration, while Chinese institutions are more inclined to cooperate with domestic partners within China.

**Table 2:** Top 10 most published institutions.

**Rank**	**Institute**	**Country**	**Count (%)**	**Citations**	**Average article citations**	**Centrality**	**H-index**
1	Harvard University	USA	112 (6.9%)	6,013	54	0.11	37
2	Institut National de la Sante et de la Recherche Medicale (Inserm)	France	91 (5.6%)	2,873	31.57	0.07	28
3	University of Liege	Belgium	91 (5.6%)	5,768	63.38	0.09	38
4	Assistance Publique Hopitaux Paris (APHP)	France	82 (5%)	3,507	42.77	0.06	26
5	Massachusetts General Hospital	USA	69 (4.2%)	3,403	49.32	0.03	29
6	University of Lausanne	Switzerland	69 (4.2%)	4,315	62.54	0.03	33
7	Center Hospitalier Universitaire Vaudois Chuv	Switzerland	68 (4.1%)	4,070	59.85	0.07	32
8	Harvard Medical School	USA	68 (4.1%)	4,605	67.72	0.06	31
9	Johns Hopkins University	USA	61 (3.7%)	2,366	38.79	0.12	26
10	Sorbonne Universite	France	61 (3.7%)	2,738	44.89	0.01	23

### 3.3 Journal analysis

The research results mentioned in this paper were indexed in 427 journals. The top 10 journals in terms of publications are shown in Table 3. *Clinical Neurophysiology* published the most articles (91, 5.6%). The journal with the highest total number of citations was *Neurology* (3,736), with an average of 138.37 citations per article. *Clinical Neurophysiology* had the highest h-index, indicating a prominent position and wide impact in the field. Among the top 10 journals by publication volume, *Neurology* had the highest impact factor, which is particularly interesting because of the relatively small number of articles, but the highest total and average number of citations per article, demonstrating the exceptional quality and broad influence in clinical neuroscience.

**Table 3:** Top 10 highly productive journals.

**Rank**	**Journal**	**Count (%)**	**Citations**	**Average article citations**	**H-index**	**IF (2023)**	**JCR (2023)**
1	Clinical Neurophysiology	91 (5.6%)	3,667	40.3	36	3.7	Q1
2	Neurocritical Care	68 (4.1%)	1,461	21.49	20	3.1	Q2
3	Journal Of Clinical Neurophysiology	60 (3.7%)	1,834	30.57	23	2.3	Q3
4	Resuscitation	48 (2.9%)	1,772	36.92	21	6.5	Q1
5	Frontiers in Neurology	42 (2.6%)	398	9.48	10	2.7	Q2
6	Frontiers in Neuroscience	35 (2.1%)	367	10.49	11	3.2	Q2
7	Clinical EEG and Neuroscience	33 (2%)	470	14.24	13	1.6	Q3
8	Brain Sciences	29 (1.8%)	209	7.21	9	2.7	Q3
9	Frontiers in Human Neuroscience	29 (1.8%)	1,030	35.52	16	2.4	Q2
10	Neurology	27 (1.6%)	3,736	138.37	19	7.7	Q1

**Figure 4:**
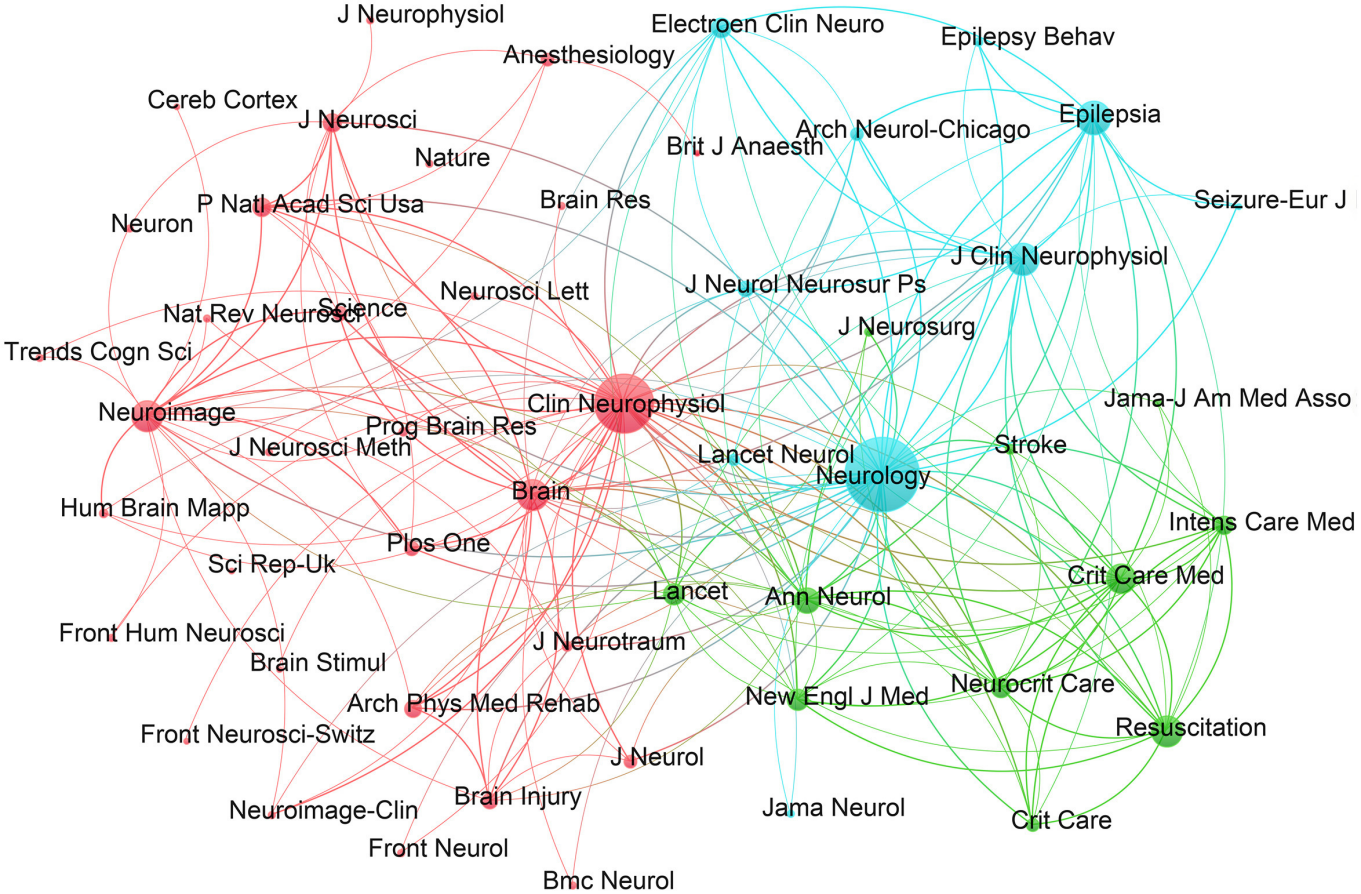
Journal co-citation network clustering map. The nodes of larger size indicate higher importance, and the connecting lines between the nodes show that the journals have been cited by the same source.

A clustering diagram of a co-citation network is presented in Figure 4. The co-citation frequency serves as an important index for measuring the influence of a journal. As evidenced in the diagram, *Clinical Neurophysiology* and *Neurology* are located at the center of the clustering. Both are Q1 journals with outstanding academic impact.

**Figure 5:**
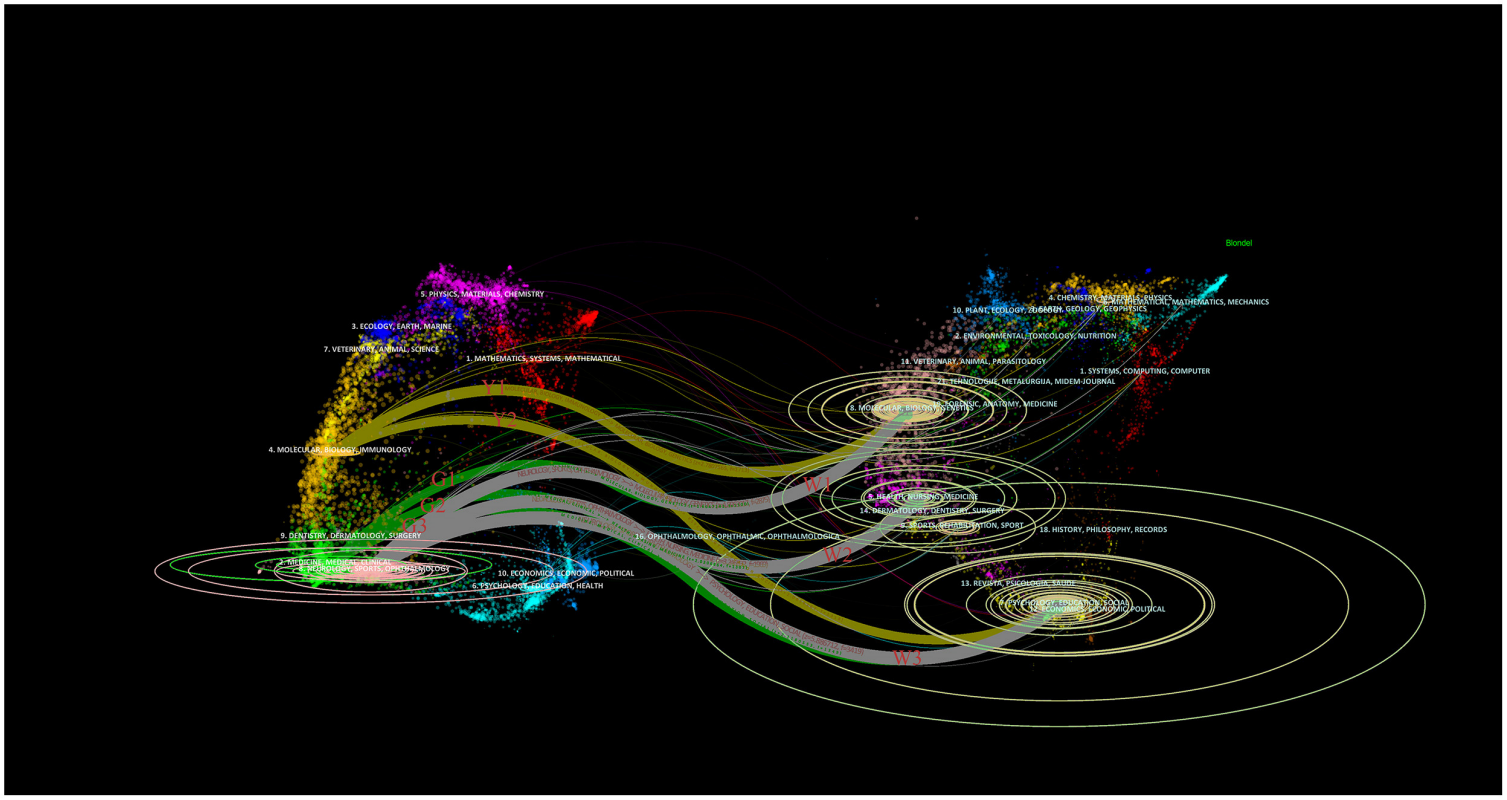
A dual–map overlay of journals. The diagram depicts the citing journals on the left, the cited journals on the right, and the connecting lines that illustrate the citation relationships. The two yellow paths are labeled Y1 and Y2, the three green paths are labeled G1, G2, and G3, and the three white paths are labeled W1, W2, and W3.

The double plot overlay presented in Figure 5 reveals mutual differences and connections between multiple layers of data. The size of the circles in the figure indicates the frequency with which the journal is cited. The larger the circle, the higher the citation frequency. The yellow pathway (Y1, Y2) shows that research in molecular/biological/immunological disciplines is often influenced by molecular/biological/genetics (*z* = 2.78, *f* = 1,713, Y1) and psychology/education/sociology (*z* = 2.06, *f* = 1,318, Y2). The green pathway (G1, G2, G3) indicates that pharmacy/medicine/clinical disciplines are influenced by molecular/biological/genetics (*z* = 1.66, *f* = 1,100, G1), health/nursing/medicine (*z* = 2.03, *f* = 1,301, G2), and psychological/educational/social (*z* = 2.12, *f* = 1,349, G3). Lastly, the white pathway (W1, W2, W3) shows that neurology/kinesiology/ophthalmology is influenced by molecular/biological/genetics (*z* = 4.90, *f* = 2,875, W1), health/nursing/medicine (*z* = 3.2, *f* = 1,969, W2), and psychology/education/social (*z* = 5.89, *f* = 3,419, W3).

### 3.4 Author analysis

**Figure 6:**
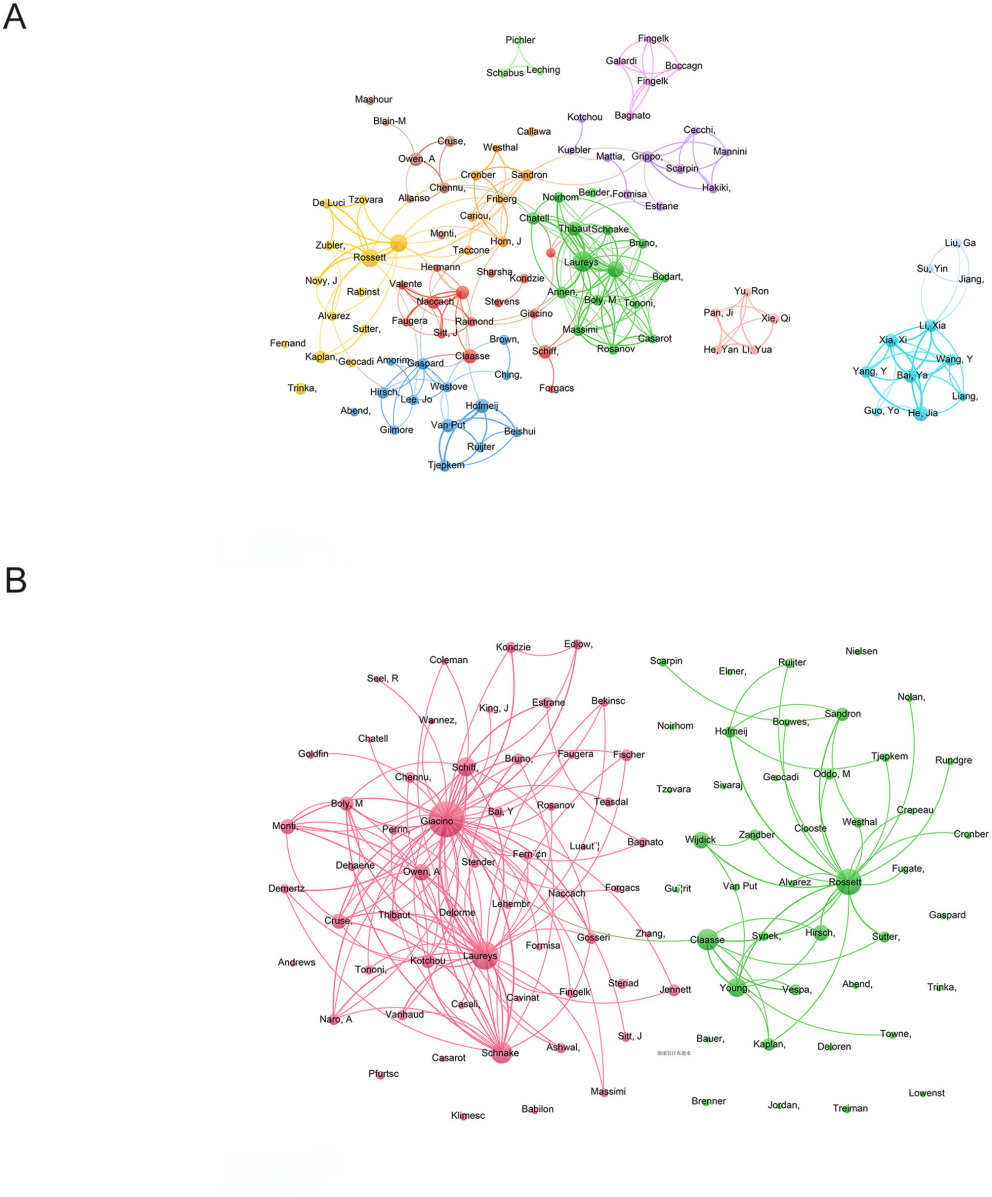
**(A)** Co-authored web. Each dot represents an author and the size of the dot indicates the number of publications by that author. The lines connecting the dots represent cooperation among authors and the thickness of the line corresponds to the frequency of cooperation. Different colors are used to distinguish between author teams. **(B)** Co-citation cluster analysis. When two authors are cited together by a third author, they have a co-citation relationship. A higher frequency of co-citation suggests a closer academic relationship between the two authors. The size of the nodes reflects the number of articles, while the lines indicate citation relationships between them.

In total, 6,956 authors published articles about the use of EEG for assessment of DoC. Of these, 202 authors published six or more articles. The top 10 authors in terms of output and the top 10 with the highest number of co-citations are listed in Table 4. Maps depicting a co-authorship network, co-citation clusters, and co-citation trends are provided in Figures 6A, B, respectively.

**Table 4:** Top 10 authors with the most publications and most co-cited authors.

**Rank**	**Count (%)**	**Author**	**Country**	**Citations**	**Average article citations**	**H-index**	**Co-cited author**	**Co-citations**
1	75 (4.6%)	Steven Laureys	Belgium	5,213	69.51	34	Joseph Giacino	1,046
2	53 (3.2%)	Andrea O. Rossetti	Switzerland	3,963	74.77	30	Steven Laureys	740
3	47 (2.9%)	Olivia Gosseries	Belgium	2,800	59.57	27	Andrea O. Rossetti	663
4	43 (2.6%)	Mauro Oddo	Switzerland	3,230	75.12	29	Jan Claassen	502
5	33 (2%)	Jeannette Hofmeijer	Netherlands	1,243	37.67	18	Caroline Schnakers	495
6	32 (1.9%)	Jan Claassen	USA	2,844	88.88	19	Nicholas D Schiff	431
7	32 (1.9%)	Jiang He	China	464	14.5	14	James B Young	431
8	31 (1.9%)	Lionel Naccache	France	2,173	70.1	21	Eelco F M Wijdicks	346
9	30 (1.8%)	Michel J.A.M. van Putten	Netherlands	1,307	43.57	19	Adrian M Owen	334
10	29 (1.8%)	Antonello Grippo	USA	622	21.45	15	Damian Cruse	309

The top three highest contributors in terms of the number of publications were Steven Laureys (75, 4.6%), Andrea O. Rossetti (53, 3.2%), and Olivia Gosseries (47, 2.9%). Steven Laureys, who leads in total citations and h-index, has been instrumental in advancing the establishment and refinement of diagnostic criteria for a minimally conscious state, exploring the natural history of vegetative and minimally conscious states, and developing therapeutic approaches to improve the quality of life for patients with DoC. Additionally, his outstanding contributions have significantly raised awareness and understanding of the field of neuroscience research among the public and healthcare providers (24–26).

A clustering diagram of co-authorship is provided in Figure 6A. Notably, cooperation was strong within the teams, but limited between teams. This is particularly noticeable concerning China, which seems to have minimal communication and cooperation with foreign teams, indicating the importance of fostering collaboration between teams. A lack of connection among teams may restrict overall effectiveness and innovation, while sharing resources across different teams can elevate research standards.

Citation of the works of two or more authors creates a co-citation relationship that reflects a close connection among scholars. Co-citations with greater frequency indicate stronger scholarly cooperation. As depicted in Figure 6B, Joseph Giacino had the most citations (1, 046), followed by Steven Laureys (740), and Andrea O. Rossetti (663).

### 3.5 Literature analysis

The top 10 most frequently cited articles are listed in Table 5. Of these 10 articles, four were reviews and six were original research. The most cited article was a systematic review published by Wijdicks et al. in *Neurology* that aimed to evaluate the prognosis of comatose survivors undergoing cardiopulmonary resuscitation after cardiac arrest (27). The second most cited article, which was authored by Claassen et al. and also published in *Neurology*, assessed subclinical seizures and unexplained decreased levels of consciousness (28). The third most cited article, which was a review authored by Brown et al. and published in the *New England Journal of Medicine* in 2010, focused on the clinical and neurophysiological features of general anesthesia, sleep, and coma by exploring the relationship between these states and the unconscious state, emphasizing the neural mechanisms of the unconscious state induced by selective intravenous anesthetics (29).

**Table 5:** Top 10 most cited publications.

**Rank**	**Author**	**Doi**	**Publication year**	**Citations**	**Source**	**Document type**
1	Eelco F. M. Wijdicks	10.1212/01.wnl.0000227183.21314.cd	2006	912	Neurology	Review
2	Jan Claassen	10.1212/01.wnl.0000125184.88797.62	2004	790	Neurology	Article
3	Emery N. Brown	10.1056/NEJMra0808281	2010	762	New England Journal of Medicine	Review
4	Damian Cruse	10.1016/S0140-6736(11)61224-5	2011	452	Lancet	Article
5	M. A. Bruno	10.1007/s00415-011-6114-x	2011	430	Journal of Neurology	Article
6	Fabio Ferrarelli	10.1073/pnas.0913008107	2010	366	Proceedings of the National Academy OF Sciences of the United States of America	Article
7	Halasz Peter	10.1111/j.1365-2869.2004.00388.x	2004	365	Journal of Sleep Research	Review
8	S. T. Herman	10.1097/WNP.0000000000000166	2015	361	Journal of Clinical Neurophysiology	Review
9	Patrick Fuller	10.1002/cne.22559	2011	353	Journal of Comparative Neurophysiology	Article
10	Melanie Boly	10.1126/science.1202043	2011	342	Science	Article

**Figure 7:**
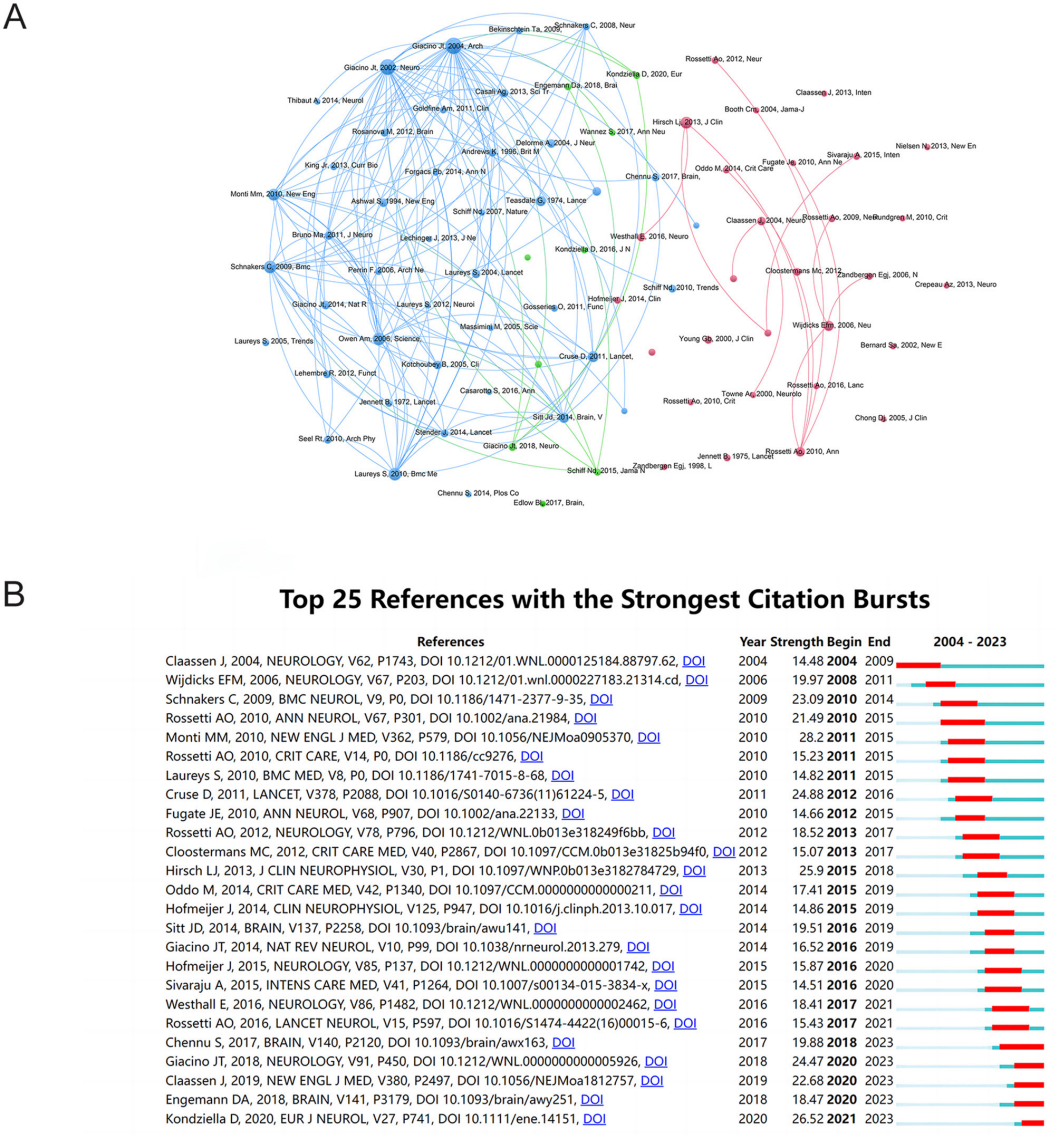
**(A)** Clustering map of co-cited literature. Different colors represent various clusters, the size of the dots indicates the number of co-citations, and the connecting lines represent citation relationships among the articles. **(B)** Top 25 references with the strongest citation bursts. The blue lines represent time intervals and the red lines represent periods with a sudden increase in the frequency of citations to that publication. The publications were ranked according to the burst strength. The higher the heat of the publication, the larger the burst strength.

Co-cited literature refers to two or more articles that were cited at least once in a later publication, demonstrating the influence of mutual citations. Analysis of this relationship can reveal common concerns within the discipline. In total, 197 articles were co-cited more than 35 times, resulting in three clusters (Figure 7A). These clusters are centered on a 2004 publication by Giacino et al. in the *Archives of Physical Medicine and Rehabilitation* (30), a 2002 publication by Giacino et al. in *Neurology* (25), and a 2013 publication by Hirsch et al. in the *Journal of Clinical Neurophysiology* (31).

The 10 most frequently co-cited papers are listed in Table 6. Two articles with Giacino as the first author are ranked first and second, with 311 and 295 co-citations, respectively. In 2002, Giacino et al. proposed the establishment of diagnostic criteria for a minimally conscious state and a consensus-based case definition that incorporates behavioral diagnostic criteria to facilitate future empirical research. In 2004, Giacino and colleagues introduced the Coma Recovery Scale-Revised, a valuable tool for measuring and diagnosing DOC, particularly in assessing the recovery of comatose patients. This scale can be conducted by experienced examiners to distinguish between UWS and MCS, as a reliable assessment of the patient's condition (25, 30). A 2,206 study by Owen et al. used functional MRI to monitor the brain activity of a vegetative 23-year-old female and found that the patient exhibited significant neural responses when listening to verbal utterances, indicating conscious activity. The results of the experiment provide a potential method for detecting the conscious activities of patients in different states. Both Giacino and Owen are dedicated to exploring diagnostic, assessment, and therapeutic approaches to impaired states of consciousness, such as a minimally conscious state, by elucidating the relationship between DoC and brain structure and function, as well as differentiating the various types of DoC using neuroimaging techniques, such as MRI and positron emission tomography, and electrophysiological studies with EEG. They also emphasize the need for standardization and international cooperation in the field, and have played significant roles in the development of international guidelines (32–34). These highly co-cited articles have introduced innovative diagnostic tools and methods for DOC, advanced the development of technology and offered significant scientific evidence and resources for clinical practice.

**Table 6:** Top 10 most co-cited publications.

**Rank**	**Author**	**Doi**	**Publication year**	**Co-citations**	**Source**	**Document type**
1	Joseph Giacino	10.1016/j.apmr.2004.02.033	2004	311	Archives Of Physical Medicine And Rehabilitation	Article
2	Joseph Giacino	10.1212/WNL.58.3.349	2002	295	Neurology	Editorial Material
3	Adrian M Owen	10.1196/annals.1417.018	2006	187	Science	Article
4	Steven Laureys	10.1186/1741-7015-8-68	2010	190	BMC Medicine	Article
5	Caroline Schnakers	10.1186/1471-2377-9-35	2009	188	BMC Neurology	Article
6	Martin Monti	10.1056/NEJMoa0905370	2010	172	New England Journal of Medicine	Article
7	Damian Cruse	10.1016/S0140-6736(11)61224-5	2011	156	Lancet	Article
8	Jacobo Sitt	10.1093/brain/awu141	2014	138	Brain	Article
9	M. A. Bruno	10.1007/s00415-011-6114-x	2011	104	Journal of Neurology	Article
10	Adenauer Casali	10.1126/scitranslmed.3006294	2013	97	Science Translational Medicine	Article

The bursts of literature citations were visualized using CiteSpace (Figure 7B). The initial citation burst occurred in a research article published in 2004, which outlines the significance of continuous EEG monitoring to detect unusual seizures in critically ill patients (28). In 2010, an article titled “Willful Modulation of Brain Activity in Disorders of Consciousness” garnered intense interest, peaking at 28.03. The article discussed the use of functional MRI to assess the ability of patients to generate specific brain responses while performing mental imagery tasks. This technique, developed by Monti et al., confirmed the consciousness and cognitive abilities in a small subset of patients in a vegetative or minimally conscious state (35). In 2021, new references continued to contribute to this area of research (4).

### 3.6 Keyword analysis

The top 25 keywords with the highest co-occurrence frequency and centrality are listed in Table 7. The top five keywords with the highest co-occurrence frequency were “vegetative state” (334), “EEG” (286), “coma” (260), “disorders of consciousness” (189), and “recovery” (180). The 25 most common keywords were interconnected, which reflected the causes of DoC, the different levels of DoC from total unconsciousness to partial awareness, patient prognosis, and the medical tools and methods used to evaluate, monitor, and treat patients with DoC. In 2010, Laureys et al. proposed the concept of UWS (80) and recommended renaming the VS (334) to more accurately reflect the patient's condition (36, 37). The term VS can mislead the public about a patient's potential awareness. Many patients may actually exhibit hidden awareness, such as cognitive motor dissociation, which can be detected through EEG. The definition of UWS more accurately reflects the patient's clinical manifestations—specifically, being awake without exhibiting behavioral responsiveness. This terminology provides a clearer understanding of the patient's physical and psychological state, avoiding the oversimplification of labeling the patient as completely unconscious. Academia is increasingly adopting the term UWS to better capture the complexity of the patient's condition and their potential for recovery (38, 39). The top five keywords with the highest centrality rankings were “detecting awareness” (0.19), “management” (0.18), “absence status” (0.18), “auditory evoked potentials” (0.18), and “cardiopulmonary resuscitation” (0.16). These keywords all focused on assessment, management, and treatment of patients with DoC using various methods and techniques to enhance quality of life and aid recovery.

**Table 7:** Top 25 most frequent keywords and highest centrality keywords.

**Rank**	**Frequency**	**Keywords**	**Centrality**	**Keywords**
1	334	Vegetative State	0.19	Detecting awareness
2	286	EEG	0.18	Management
3	260	Coma	0.18	Absence status
4	189	Disorders of Consciousness	0.18	Auditory evoked potentials
5	180	Recovery	0.16	Cardiopulmonary-resuscitation
6	178	Cardiac arrest	0.16	Convulsive status epilepticus
7	176	Minimally conscious state	0.15	Predictive-value
8	131	Traumatic brain injury	0.14	Mismatch negativity
9	129	Nonconvulsive status epilepticus	0.14	Anoxic coma
10	123	Status epilepticus	0.13	Intensive care unit
11	116	Consciousness	0.12	Prognostic value
12	103	Therapeutic hypothermia	0.12	Evoked-potentials
13	100	Brain	0.11	Connectivity
14	95	Awareness	0.11	Triphasic waves
15	89	Connectivity	0.1	Postanoxic coma
16	84	State	0.1	Persistent vegetative state
17	80	Prognosis	0.1	fMRI
18	80	Unresponsive wakefulness syndrome	0.1	Delirium
19	75	Seizures	0.1	Architecture
20	73	Brain injury	0.09	Event-related potentials
21	72	Prognosis value	0.09	Bispectral index
22	71	Patterns	0.09	Cerebral cortex
23	67	Event-related potentials	0.08	Anesthesia
24	67	Functional connectivity	0.08	Effective connectivity
25	66	Intensive care unit	0.08	Communication

**Figure 8:**
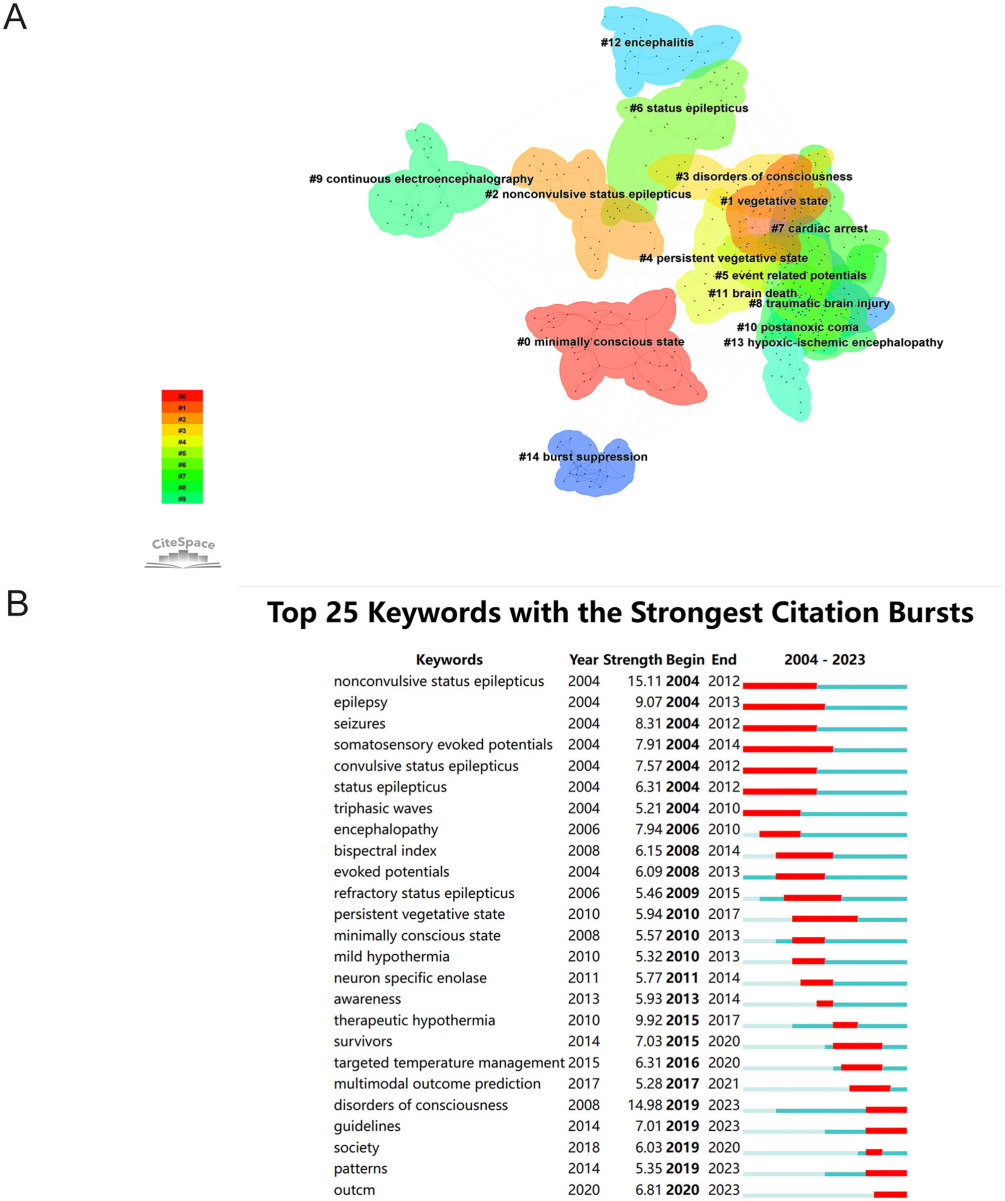
**(A)** Keyword clustering network. **(B)** Top 25 keywords with the strongest citation bursts. Blue lines indicate time intervals. Red lines indicate periods of sudden increases in citation frequency.

Keyword clustering analysis groups keywords in the literature to identify different research topics and reveal the evolution of knowledge structure and research directions within a field. A cluster network diagram of keywords associated with the use of EEG for assessment of DoC was created with CiteSpace software to visualize the knowledge structure in this field (Figure 8A). Clustering with the Latent Semantic Indexing algorithm yielded 15 cluster labels with a mean silhouette value of 0.8975 (>0.7) and a modularity *Q* value of 0.781 (>0.3), demonstrating good homogeneity among clusters and significant clustering results. The five largest clusters were #0 Minimally conscious state, #1 Vegetative state, #2 Nonconvulsive status epilepticus, #3 Disorder of consciousness, and #4 Persistent vegetative state. These clustering labels represent the main themes of the research field and can be roughly categorized into three groups. The first group [#0, #1, #2, #4, #6, #14] focused on the clinical manifestations and diagnosis of DoC. The second group [#5, #9] was primarily concerned with techniques and theories for diagnosis of DoC. The third group [#7, #8, #10, #11, #12, #13] mainly focused on DoC caused by specific diseases. The largest cluster #0 was associated with a minimally conscious state, which included cardiac arrest, complex partial status, continuous wavelet, brain death, and quantitative EEG. These findings indicate a deepening of research on various states of consciousness in recent years.

A “burst” in keywords refers to a sudden increase in the frequency of keyword occurrences, which usually indicates cutting-edge topics in a field at different times and can also be used as an indicator of research trends. In this study, CiteSpace software was used to identify the 25 most explosive emergent words, which are listed in Figure 8B, where the blue lines represent the time interval and the red lines represent the period when the keyword appeared. The keywords with the highest outbreak values, aside from DoC, were “nonconvulsive status epilepticus” (15.11), which emerged as the earliest notable keyword between 2004 and 2023, followed by “therapeutic hypothermia” (9.92) and “epilepsy” (9.07). This trend indicates that monitoring and treatment for DoC is a prominent area of research.

## 4 Discussion

### 4.1 Basic information

The use of EEG for assessment of DoC has gradually expanded in recent years, as an indispensable tool from early basic research to modern advanced analytical techniques. The use of EEG for assessment of DoC focuses on diagnosis, evaluation, and prognosis of the potential of patients to regain consciousness. Analysis of EEG data allows for assessment of the state of consciousness to select an appropriate treatment plan. At present, EEG for assessment of DoC is a significant research area with a promising future.

Over the past 20 years, research on the application of EEG for assessment of DoC has shown fluctuating growth in both the number and quality of publications. Several countries and institutions have made significant contributions to this area of research at the international level. The United States and Italy are regarded as pioneers. Both the United States and Italy excel in terms of total citations and the h-index. The high-quality publications from these two countries have made significant contributions to the field, far surpassing other countries by a considerable margin. China is ranked third in terms of the number of publications, showing explosive growth in 2022 and 2023. However, due to a relatively late start and less cooperation with the international community, China is less influential than the United States and Italy. Hence, strengthening cooperation with other countries and institutions, and commitment to high-quality studies will improve the influence of research conducted in China.

Analysis of the most prolific authors can help to identify the core topics in the field of neuroscience research. As the most academically influential author internationally, Steven Laureys from Belgium had the highest number of publications and citations, with primary research interests focused on the mechanisms and EEG-based assessment of DoC, pain management, and neuromodulation therapy (40–43). Through in-depth analysis of EEG signals, Laureys used the DoC-Forest tool to combine various EEG data, including alpha-band power, theta-band connectivity, and time-series complexity, to assess the state of consciousness, which significantly reduced the impact of different EEG configurations and experimental protocols on the distribution and performance of EEG markers to identify several atypical signs of consciousness that may be overlooked by traditional clinical assessment methods. Since there are limitations to processing of a single EEG signal, Laureys suggested a multimodal assessment strategy that incorporates other neuroimaging and neurophysiology techniques for more comprehensive assessment of the state of consciousness. Also, Laureys has been focused on innovative auxiliary communication and control channels to develop a brain-computer interface system based on electroencephalographic activity. These interfaces use control signals, such as event-related potentials, steady-state visual evoked potentials, and sensory-motor rhythms, to provide DoC patients with a new means of communication. These studies not only provide new diagnostic and therapeutic tools for DoC patients, but also offer an important scientific basis for understanding the neural underpinnings of consciousness (44–46).

*Clinical Neurophysiology* had the highest number of publications and the highest h-index, while *Neurology* had the lowest number of publications and the highest number of co-citations.

### 4.2 Research hotspots and future trends

Analysis of the keywords was conducted to identify research trends and hotspots in the field of neuroscience research. The most frequently occurring keywords and those with the highest centrality are shown in Table 7. Most of these keywords (“coma,” “vegetative state,” “minimally conscious state,” “EEG,” “prognosis,” “monitoring,” and “status epilepticus”) were related to DoC and organized into 15 clusters. The first five clusters were focused on the diagnosis and evaluation of different states of DoC. From 2004 to 2013, the emergent terms “nonconvulsive status epilepticus,” “seizure,” “convulsive status epilepticus,” “encephalopathy,” and “epilepsy” had the highest intensity, indicating that attention was directed toward the use of EEG for monitoring and differentiation of different types of seizure states. Terms that emerged between 2008 and 2014, such as “evoked potentials,” “bispectral indices,” and “neuron-specific enolase,” reflected focus on improvements to EEG techniques for assessment of DoC. Between 2015 and 2023, emergent terms included “hypothermia,” “target temperature management,” “guidelines,” and “society,” demonstrating a greater emphasis on the use of EEG in combination with other techniques for management of DoC. As research progresses, an increasing number of clinical guidelines are integrating EEG into the diagnosis of DOC. These standardized protocols enhance diagnostic consistency across various medical institutions.

Comprehensive analysis of the keywords was conducted to clarify the application of EEG for diagnosis, monitoring, and prognosis of DoC over the past two decades. EEG has been more commonly applied for the diagnosis and prognosis of DoC during this period, as several research groups have been concerned with accurate differentiation of the states of DoC, particularly the UWS and MCS. Although these two states may present similar clinical manifestations, their underlying neurological functions and prognoses differ significantly. Therefore, accurately distinguishing between them is crucial for developing effective treatment plans and predicting patient outcomes. Researchers utilize event-related potential-based EEG examinations to evaluate the state of consciousness in patients with severe consciousness disorders. This approach allows for the differentiation of patients based on their levels of consciousness by detecting event-related potential components related to consciousness, such as P300, N400, and P600. In several clinical trials, patients exhibiting the P300 component typically demonstrated a gradual improvement in their state of consciousness within the MCS, the P600 component is typically absent in patients with UWS (47–50). The N400 component is widely used to evaluate language processing and cognitive function. Monitoring changes in the N400 can offer insights into a patient's potential for subconscious recovery (51). You et al. (52) recorded EEG responses from 20 healthy subjects under three conditions: active mode (with visual and auditory stimuli), passive mode (where subjects observed brain activity without responding to stimuli), and breathing mode (which involved focusing on breathing to minimize external stimuli processing). Analyzing various characteristics of EEG responses to different modes and stimuli, the researchers found that the amplitude of the event-related potential in the respiratory mode was significantly reduced, demonstrating greater distinguishability compared to the active counting mode. This finding provides new insights for more sensitive and accurate detection of consciousness. Wutzl et al. (53) observed that the alpha wave power in patients with MCS tends to be higher than in those with UWS, while delta wave power is generally lower in MCS patients. Additionally, MCS patients demonstrate better performance in weighted symbol cross-entropy and transfer entropy across theta, delta, and alpha frequency bands, indicating that their brain activity is more complex. In patients with UWS, functional connectivity is typically lower, particularly between the frontal and parietal lobes. In contrast, patients with MCS exhibit enhanced interactions between local and distant cortical networks. These differences may reflect the fluctuations in consciousness that MCS patients experience, which are often underestimated in behavioral assessments.

The development of signal processing technology for EEG is accelerating at an ever faster pace. Noise and artifacts in EEG signals have long posed challenges for analysts, but recent advancements in EEG signal processing technology have significantly improved this situation. Versaci and La Foresta (54) explored the effectiveness of fuzzy logic techniques, particularly intuitionistic fuzzy systems, in eliminating both internal and external noise from EEG signals. These methods not only enhance the accuracy of artifact identification and removal but also integrate seamlessly with artificial intelligence techniques to advance EEG analysis. Wang et al. (55) observed changes in the EEG microstates of patients with DOC during hyperbaric oxygen therapy. EEG microstates, which serve as biomarkers—including mean microstate duration, total time coverage ratio, and global explained variance—are crucial for diagnosing and assessing the prognosis of patients with DOC. The study employed real-time EEG recording technology to compare and analyze the microstate indicators of patients before and after hyperbaric oxygen therapy. The results demonstrated significant changes in these EEG microstate indicators corresponding to the patients' state of consciousness during treatment. This provides an important electrophysiological measure for assessing consciousness and may offer an objective basis for the precise treatment of patients with DOC. Overall, these findings underscore the importance of advanced EEG signal processing and analysis techniques in improving our understanding and treatment of DOC.

A variety of technologies are being developed for integration with EEG. Ahmad et al. pointed out that EEG offers high temporal resolution and can capture transient changes in EEG activity (56). The high spatial resolution of functional MRI can accurately localize activity in different brain regions (57). The combination of these two techniques can gather information about both EEG activity and blood oxygen level-dependent signals to clarify the working mechanisms of the brain. A study investigated the use of EEG and functional magnetic resonance imaging to assess states of consciousness in patients with multiple brain injuries and DOC. During a hand squeeze task, some patients exhibited contralateral event-related potentials in the theta band, mirroring patterns seen in healthy individuals. This suggests that combining various tasks and neuroimaging techniques can enhance the accuracy of detecting states of consciousness in patients with DOC (58). TMS-EEG technology has proven to be a reliable method for differentiating between conscious and unconscious patients. The precise stimulation capabilities of EEG combined with TMS can quantify the brain's complexity across different states, making it a powerful tool for studying cortical excitability and connectivity (10). Additionally, brain-computer interface (BCI) technology shows promise for facilitating communication and monitoring the state of consciousness in individuals with DOC. BCI systems that leverage vibration and P300 signals can be used to assess the state of consciousness and provide an innovative communication method for unresponsive patients (59). With the ongoing optimization application of new technologies, EEG is poised to play an increasingly significant role in future clinical practice.

In patients with DOC, EEG can be employed for the rapid diagnosis, prediction, and monitoring of seizures in emergency rooms and intensive care units. For instance, nonconvulsive epilepsy and epilepsy share similar clinical symptoms, but there are notable differences in causes and treatment options (60). Advancements in EEG signal processing and machine learning algorithms have facilitated theoretical studies for the detection and prediction of epileptic seizures (61). These developments offer the potential for more timely interventions for patients, thus improving treatment outcomes and quality of life. The EEG biomarkers functional connectivity and dominant frequency are reportedly predictive of the clinical outcome of non-traumatized patients, at least to some extent (62). The combined application of these markers can more accurately assess the recovery potential of patients, thereby providing a basis for individualized treatment and intervention. Rubinos et al. reported that specific EEG patterns are predictive of long-term functional and cognitive outcomes, as well as recovery of consciousness after acute brain injury (63). This association is particularly useful to predict outcomes of critically ill patients after ischemic stroke, intracranial hemorrhage, subarachnoid hemorrhage, traumatic brain injury, anaerobic brain injury, and metabolic encephalopathy.

The emergence of the keywords “targeted temperature,” “management,” “multimodal outcome prediction,” “guidelines,” “society patterns,” and “outcomes” have significantly increased in recent years, suggesting that future research may focus on prognostic assessment of DoC, monitoring treatment effects, and integrated multimodal assessment. EEG technology holds significant promise for clinical applications in the treatment of DOC. The spontaneous oscillation state of the EEG, along with ultra-long cycle rhythm differences and other characteristics, can more accurately assess the level of consciousness and inform clinical decision-making. By analyzing EEG signals, it is possible to determine whether a patient has residual consciousness, which is critical for developing an individualized treatment plan. EEG is not only valuable for diagnosis but also for predicting the prognosis and rehabilitation of patients with consciousness disorders, particularly in assessing their short-term outcomes. In extreme cases, EEG can identify potential markers of consciousness and detect its presence even in the absence of behavioral evidence, offering a new diagnostic tool for patients who cannot be effectively evaluated by traditional methods. Furthermore, EEG can be combined with other diagnostic tools to provide a more comprehensive evaluation of brain network recovery and changes in metabolic patterns, thereby offering new insights for treatment. There is a need to further optimize classification algorithms and enhance the accuracy of data interpretation. Future research will focus on addressing these issues to more effectively utilize EEG technology in clinical practice for evaluating the consciousness states of patients with these disorders. With ongoing technological innovation and research, EEG is expected to become an essential tool for assessing and treating patients with consciousness disorders, ultimately aiding efforts to improve patient prognosis and quality of life.

## 5 Challenges and limitations

In this study, future research hotspots were predicted and new perspectives were provided. However, only English-language publications retrieved from the Web of Science Core Collection database were included for analysis. This single data source and the exclusion of non-English-language literature may have led to incomplete results, as certain smaller or emerging areas of research might not have been adequately indexed. We plan to broaden our literature search in future work by including additional international databases such as PubMed, Scopus, and Google Scholar. We will also consider incorporating relevant articles published in other languages to provide a more comprehensive view of the research landscape. Subjective bias may be introduced during the literature screening process, leading to research results that lack objectivity. Bibliometric analysis relies on published literature, which may not adequately capture emerging research trends and hotspots. Existing bibliometric tools and techniques face challenges related to scalability and automated integration, as data must often be manually exported from providers and imported into the tools. This manual process adds complexity and increases the difficulty of analysis. The methods of bibliometric analysis are somewhat rigid and may not fully capture the actual impact of a particular study, potentially resulting in the oversight of the quality and innovativeness. We intend to enhance our findings in the future by conducting qualitative assessments of key studies to underscore their significance and contributions to the field. This approach will provide a more comprehensive understanding of the literature and its impact on clinical practice.

## 6 Conclusion

The visualization tool CiteSpace V6.2 R6 and VOSviewer software were utilized to assess current and future trends of the application of EEG for assessment of DoC to identify the emerging areas of research and provide fresh perspectives for future studies. Since 2005, research on the use of EEG for assessment of DoC has increased in quality and quantity. The main current issues in the field of DoC are the accurate diagnosis of the level of consciousness and the identification of early residual consciousness. Emerging trends focus on exploring the neural mechanisms of recovery of consciousness and the development of effective treatment strategies for patients with DoC, especially neuromodulatory approaches. Overall, these findings were based on analyses of countries, institutions, authors, keywords, and references. These findings provide a more comprehensive understanding of the field and will help to guide future research.

## Data Availability

The original contributions presented in the study are included in the article/supplementary material, further inquiries can be directed to the corresponding authors.
